# Arsenic in Beer

**Published:** 1906-02-10

**Authors:** 


### Arsenic in Beer.

The recent report by Dr. Collingridge upon the occasional, or even frequent, presence of minute quantities of arsenic in beer, although it points to an evil which seems to be very small in comparison with that which occasioned the extensive and fatal outbreak of disease in the North of England a few years ago, is yet by no means to be disregarded. The word "beer," in successive periods of English history, has been used to express a great variety of compounds; and, in the reign of Queen Elizabeth, there is said to have been an enactment forbidding the "adulteration" of beer with hops. At the present time the chief source of danger to which consumers are exposed appears to arise from the employment of malt which is liable to receive small quantities of arsenic in the process of kiln drying, the mineral being volatilised from the fuel, or sometimes being projected from it in particulate form. We are not sufficiently acquainted with the mechanism of malt drying to say whether it could not be accomplished on floors heated from below by some less objectionable means than those which appear now to be in use, and which, it is safe to presume, have at least the advantage of cheapness. The problem does not seem to be an insoluble one; and it would perhaps be worth the consideration of the strong "labour" element in the new House of Commons whether they should not press for legislation to protect the favourite beverage of their constituents from an admixture so likely to be injurious to consumers. A small enhancement of price, if unavoidable in the presence of a demand for wholesomeness, would scarcely be objected to by any even moderately intelligent purchaser, and the high probability is that any really necessary enhancement would be too small to affect the retail buyer, and would only be felt, if anywhere, as a fractional diminution of the profits of the producer. It is unfortunately certain that the well-founded arsenical scare in the North of England had, while it lasted, the effect of increasing the demand for spirits rather than the practice of temperance; and it is undoubtedly a national interest that the favourite beverage of the working classes should at least be free from any injurious properties other than those which are necessarily associated with its alcoholic foundation. Even if we do not go so far as those who assert that alcohol, in all its forms and combinations, is injurious to the healthy human subject, we must at least concede that it should not be sold in combination with matters possibly still more injurious than itself.

At a time when the infinitely little is every day coming more and more into prominence as a factor in disease, few questions are more interesting than those which relate to influences exerted upon the economy by the continued operation of small and frequently repeated doses of some noxious material. From the time when Sir George Baker traced the so-called "Devonshire colic" to the lead linings of the vessels employed in the manufacture of cider, physicians have been familiar with the effects of lead thus gradually introduced into the system; and the arsenical poisoning by brewers' glucose, to which we have above referred, was an apt illustration of the effects of still smaller doses of a mineral poison, which displayed itself chiefly by the production of diffused neuritis. Is it not quite possible that a great many of the varied forms of nerve degeneration with which, in the present day, we are unfortunately only too familiar, may be due to the operation of the same or of analogous causes, and that the foundations of "neurasthenia," in some at least of its varied forms, might be discoverable rather by the analytical chemist than by the merely clinical observer? Recent researches appear to show that, in the human subject small and frequently repeated doses of arsenic are frequently followed by a deposition of the metal in the hair; and it might be conducive to better knowledge of many neurotic conditions if the physicians to hospitals chiefly dealing with them would regard a chemical examination of the hair of patients as one of the lines of research which should be systematically followed. If the hair receives arsenic, it may receive other poisons in the same manner; and the report of Dr. Collingridge at least suffices to show that arsenic, of the cumulative effects of which we have lately obtained conclusive evidence, is far more extensively administered to our population than, until recently, had even been conjectured.

